# Time pressure increases children’s aversion to advantageous inequity

**DOI:** 10.3389/fpsyg.2024.1390741

**Published:** 2024-06-05

**Authors:** John Corbit

**Affiliations:** Department of Psychology, St. Francis Xavier University, Antigonish, NS, Canada

**Keywords:** cooperation, fairness, intuition, deliberation, inequity aversion

## Abstract

The relative contribution of intuitive and reflective cognitive systems in cooperative decision making is a topic of hot debate. Research with adults suggests that intuition often favors cooperation, but these effects are contextually sensitive. Emerging evidence has shown that in many contexts children show a tendency toward intuitive cooperation, but research investigating these processes in children is sparse and has produced mixed findings. In the current study we investigated the influence of intuitive and reflective decision processes on children’s fairness behavior by manipulating decision time. We tested (*N* = 158) pairs of children between 4 and 10 years of age from a rural community in Canada. Children’s decisions to accept or reject allocations of candies were either made under time pressure or after a 10-s delay. We assessed the impact of decision time on children’s aversion to inequitable distributions of resources by comparing their responses to equal allocations with either disadvantageous allocations or advantageous allocations. We found that children showed a greater age-related increase in advantageous inequity aversion when decisions were made under time pressure compared to when they were made after a delay. In contrast, we did not observe a significant impact of decision time on the development of disadvantageous inequity aversion. These findings suggest that intuitive decision processes may contribute to the development of fairness concerns in middle childhood.

## Introduction

Fairness concerns are thought to guide resource allocation behavior toward mutually satisfactory outcomes, but behaving according to principles of fairness can be personally costly. The observation that humans are willing to engage in costly cooperative behavior, has led to a long-standing debate about why cooperation persists even when pitted against immediate self-interest (Tomasello and Vaish, 2013). A prominent approach that has been applied to understanding the cognitive processes that determine cooperative behavior is the dual-system model, which pits a fast intuitive system against a slow deliberate system as determinants of human decision making. While the dual system approach has gained traction as a leading theoretical model to account for cooperative decision making, there is considerable debate amongst dual system theorists about which system is dominant in cooperative decisions. One leading theoretical perspective suggests that humans are inherently selfish, but through deliberate, effortful control this tendency toward self-maximizing can be inhibited in favor of costly cooperative behaviors (Trivers, 1971; Stevens and Hauser, 2004). Alternative models propose that cooperation is intrinsically motivated (Batson and Shaw, 1991; Hoffman, 2000; Warneken and Tomasello, 2009), largely intuitive, and independent of effortful control (Rand et al., 2012; Zaki and Mitchell, 2013). Importantly, framing the question of the nature of cooperative decision making as originating from either intuitive or deliberate cognitive processes overlooks the fact that cooperation comes in many forms, and in all probability both processes are important determinants of cooperative behavior with the relative contribution varying considerably across cooperative types and contexts. As we begin to map the relative role of intuition and deliberation in cooperative decisions it is important to study their emergence and subsequent development to understand how these processes function in specific cooperative contexts, such as fairness behaviors, which are thought to play a fundamental role in the organization of human cooperative relations.

A well-established approach to assessing the contribution of intuitive and reflective decision processes on cooperative behavior is to examine the impact of decision time on cooperative behaviors. The dual-process model of decision making proposes that decisions arise from distinct cognitive systems: a fast, intuitive, or ‘hot’ system that is efficient but relies on heuristics and is susceptible to bias and predictable errors, contrasted with a slow, deliberate or ‘cold’ system that is more cognitively demanding but can be applied flexibly and rationally through deliberate control (Sloman, 1996; Gilovich et al., 2002; Kahneman, 2011; Greene, 2014; Corbit et al., 2023a). Importantly, the dual-process model is hotly debated within the behavioral sciences with some theorists supporting a single-process model where deliberate and intuitive decisions represent differences in degrees rather than qualitatively distinct systems (Kruglanski and Gigerenzer, 2018). Furthermore, others argue that forming a distinction between single- and dual-process models is intractable given current empirical evidence (De Neys, 2021). While the application of dual-process models of decision making remains a topic of debate there is substantial agreement that manipulating response time is an experimental approach that typically correlates to the two cognitive processes, with fast decisions more often recruiting an intuitive process and slow decisions more often relying on deliberate processes (Capraro, 2024).

Recently, a considerable literature has emerged that explores the relative contribution of intuitive and reflective processes on cooperative decisions. Several lines of converging evidence suggest that adults may be intuitive cooperators under specific contexts. For example, in public goods games, where adults must decide between outcomes that are self-maximizing or cooperating to provide a larger benefit to their group, decisions made under time pressure or those primed with intuitive thinking are more likely to be cooperative (Cappelletti et al., 2011; Cornelissen et al., 2011; Rand et al., 2012; but see Bouwmeester et al., 2017). Further, meta-analytic evidence suggests that intuitive cooperation is more likely to occur under *pure cooperation* conditions, where cooperation does not provide a personal benefit, and when it is unlikely to be exploited (Rand et al., 2014; Rand, 2016). In the ultimatum game, wherein participants can decide to accept or reject offers of resource and behavior closely maps to fairness preferences, a meta-analysis found that adults were more likely to reject unfair offers when intuitive decision processes were promoted (Capraro, 2024). Further, the effect of intuition on sharing decisions, as when allocating resources to a single passive recipient, may show less impact of intuitive decision processes according to recent meta-analyses that found no evidence for an impact on altruistic sharing from promoting intuitive or reflective decision processes (Fromell et al., 2020; Capraro, 2024). Together this this evidence suggests that amongst adults the impact of intuitive and reflective decision can vary considerably based on the type of cooperative decision and the context within which cooperative decisions are made.

Recently, researchers have begun to apply a dual-process model to study the development of cooperative decision making with mixed results. In one study, toddlers who were fast to provide help in response to an experimenter’s need showed a higher rate of helping over repeated trials compared to those who were slow to respond (Grossmann et al., 2020). For older children (3–7 years of age), sharing decisions that were made under time pressure tended to be more generous compared to decisions that were made after a 10-s delay (Plötner et al., 2021). Additionally, in a modified public goods game that gave children (7–12 years of age) a choice between 2 resources for themselves or 1 each for themselves and three group members (costly cooperation), children were more likely to cooperate when their decisions were made under time pressure compared to a 10-s time delay or a neutral condition where decision time was unconstrained (Corbit et al., 2023a). While these studies highlight an important role for intuitive cooperation throughout early to late childhood, another recent study reveals a different pattern when children are given a choice to share resources equally or give more to themselves. In this study children (4–9 years of age) were given a choice between an equal distribution of resources (2 each) or a selfish distribution (3 self, 1 recipient) and their decision times were recorded (Chajes et al., 2022). Young children (4–6 years of age) rarely chose the equal option, but when they did, these decisions were slower compared to the selfish ones. Older children (7–9 years of age) were more likely to choose the equal distribution, but in this case their decisions were not predicted by decision time (Chajes et al., 2022). Taken together, these findings suggest that from an early age children show a tendency toward intuitive cooperation across several cooperative contexts, but this pattern might not extend to costly fairness.

Based on the evidence reviewed thus far, the impact of intuitive decisions on the development of fairness is difficult to adjudicate due to methodological differences across previous studies examining children’s sharing decisions. For instance, while Plötner et al. (2021) found that time pressure increased generosity relative to a time delay, children in this study were given nine resources to distribute and so distributing equally was not an option. In the case of Chajes et al. (2022), children received the option of distributing resources equally, but equal decisions were also more generous as they gave the recipient 2 resources rather than 1 resource for the recipient if children chose the selfish option. Finally, Corbit et al. (2023a) found that for intuitive decisions children were more likely to give resources to their group, a decision that resulted in an equal distribution with each player receiving 1 resource, while selfish decisions resulted in an unequal distribution with the participant receiving 2 resources and the other players none. In this case cooperative decisions achieved equality but may have been aimed to ensure that the other players got something, rather than being governed by a strict preference for fairness. Thus, to clarify the impact of intuitive decisions on children’s specific concern for fairness it is important to employ a methodology that can adjudicate between a motivation to achieve fairness and other cooperative concerns, such as generosity.

A common method to assess children’s concern for fairness independently of generosity is to examine how they respond to unequal distributions of resources. Achieving fairness by discarding unequal distributions of resources demonstrates a concern for fairness that is non-generous, and so provides insight into fairness preferences that are distinct from generosity (Corbit et al., 2017). Avoidance of unequal distributions to achieve fairness has been described as an aversion to inequity (Fehr and Schmidt, 1999). Inequity aversion takes two forms; disadvantageous inequity aversion avoids receiving fewer resources than a peer, whereas advantageous inequity aversion avoids receiving more resources than a peer. When young children (4–6 years of age) are presented with fewer resources than a peer, they avoid this disadvantageous form of unfairness by rejecting those distributions, a pattern observed across societies (Blake et al., 2015). Remarkably, older children (~7–8 years of age) in some societies also reject unequal resource distributions that advantage them by giving them more than a peer (Blake et al., 2015; Paulus, 2015; Corbit et al., 2017; Kajanus et al., 2019; Li et al., 2022; Corbit et al., 2023b).

Advantageous and disadvantageous inequity aversion are likely supported by different cognitive processes. For instance, disadvantageous inequity aversion might be motivated by spite or envy toward an advantaged recipient (McAuliffe et al., 2011; Shaw and Olson, 2012) and may well represent a mild form of reactive aggression elicited by a negative emotional response to receiving less (Brosnan and de Waal, 2014). Advantageous inequity aversion signals a strong concern for other regarding fairness and may be motivated by avoiding negative social emotions, such as guilt or shame, and adherence to social norms (Blake et al., 2015; Gerdemann et al., 2022). Recent efforts to understand the cognitive mechanisms that produce children’s fairness decisions have found that distinct evaluative (Amir et al., 2023) and inhibitory (Sobel et al., 2024) processes underlie responses to advantageous and disadvantageous inequity. Importantly, although these studies have provided new insights about the cognitive factors that foster the development of fairness concerns, the role of intuitive and reflective decision processes on the development of inequity aversion remains an open question.

### Current study

In the current study we sought to assess the impact of intuitive and reflective decision processes on children’s fairness behavior. We included children between 4 and 10 years of age from a rural community in (blinded) Canada. This age range was chosen to capture the typical emergence of disadvantageous inequity aversion (4–5 years of age) and advantageous inequity aversion (7–9 years of age) amongst children developing within similar Western contexts (Blake et al., 2015; Corbit et al., 2023b). We were interested in how decision time impacted children’s responses to both advantageous inequity and disadvantageous inequity. Thus, we presented children with a modified version of the Inequity Game, developed by Blake and McAuliffe (2011). The modified version presented children with allocation decisions on a tablet, which allowed for control and accurate measurement of the time from the onset of the presentation of allocation choices to participants’ decisions (decision time). The Inequity Game presents pairs of children with allocations of resources that are either equal (1 each) or unequal. Unequal allocations are either advantageous (4 for self and 1 for recipient) or disadvantageous (1 for self and 4 for recipient), depending on the condition to which the participants are assigned. In the modified version, pairs of children (one designated as actor and one as recipient) observed the allocations on a tablet and the child designated as actor made decisions to accept or reject allocations presented by the experimenter for the duration of the game. An aversion to inequity was considered to occur when children rejected unequal offers significantly more often than equal offers.

To assess the impact of intuitive and deliberate decision processes on children’s aversion to both types of inequity, we assigned children to either a Fast or Slow Decision Time condition using a manipulation based on Corbit et al. (2023a), and then to either an Advantageous or Disadvantageous Inequity Type condition. In the Fast conditions (Fast/Advantageous, Fast/Disadvantageous) children were asked to make their decision as quickly as possible after each allocation was presented. In the Slow conditions (Slow/Advantageous, Slow/Disadvantageous) actors were asked to hold and consider their decisions after the allocation was presented until the experimenter indicated after a 10-s delay that it was time to make their decision.

As was highlighted above, there is convergent evidence to support the perspective that advantageous and disadvantageous inequity aversion are likely supported by different cognitive mechanisms, and so we consider our predictions for the effect of decision time on these types of inequity aversion separately. Children’s tendency to reject advantageous offers would provide evidence of a robust, other-regarding concern for fairness that is both costly and non-generous, whereas accepting advantageous offers would signal a motivation to self-maximize resources. In contrast, rejecting disadvantageous offers would signal a personal concern for fairness that is less costly, and might be motivated by spite rather than a strict concern for fairness (McAuliffe et al., 2011). Alternatively, accepting disadvantageous offers could signal a tendency toward generosity, as it would endow the recipient with a relative advantage over the actor. Thus, children’s behavior on advantageous trials would be particularly informative for the effect of decision time on fairness concerns (or self-maximizing), whereas behavior on disadvantageous trials would inform motivations toward generosity (or spite).

As we were principally concerned with the impact of intuitive decision processes on children’s concern for fairness our primary hypothesis (H1) predicted that time pressure would increase advantageous inequity aversion compared to time delay. Furthermore, we predicted an earlier age of emergence of advantageous inequity aversion when decisions were made under time pressure. Alternatively (H2), it is possible that costly fairness decisions rely on the inhibition of tendencies toward self-maximizing (Steinbeis, 2018; Chajes et al., 2022), in which case advantageous inequity aversion would be more likely to emerge in decisions made after a time delay compared to those made under time pressure. On disadvantageous trials it was possible that that time pressure would increase children’s tendency toward generosity (Plötner et al., 2021), in which case we would expect a reduction in the rejection of disadvantageous trials (H3). Alternatively, disadvantageous inequity aversion might depend in part on a negative emotional response to receiving fewer resources than a peer (Brosnan and de Waal, 2014), in which case time pressure may default to the reliance on this initial emotional reaction and lead to greater rejection of disadvantageous offers under time pressure relative to time delay (H4). For the sake of formalizing our predictions into testable hypotheses we have separated these predicted outcomes into four separate hypotheses, but it is important to note that they need not be mutually exclusive. Indeed, the impact of intuitive and reflective decisions processes is likely to be multi-factorial and shift over development.

## Method

### Participants

This study included a total of 158 participants sampled continuously between 4 and 10 years of age. Specifically, our sample included 48 4- to 5-year-olds (*M* = 4.69 years, 20 females), 59 6- to 7-year-olds (*M* = 6.53 years, 22 females) and 55 8- to 10-year-olds (*M* = 8.67 years, 32 females). An additional 4 participants were tested and excluded because they could not understand the procedure (*N* = 3) or said they “did not care about the game” because they do not like Skittles (*N* = 1). Our goal sample size was based on typical sample sizes using similar designs with the Inequity Game, with the goal of achieving 40 participants in each condition (Corbit et al., 2017; Gonzalez et al., 2020). Although we did not conduct an *a priori* power analysis to determine our sample size, a sensitivity analysis using G*Power version 3.1 (*α* = 0.05, 1-β = 0.80, df = 5) revealed that with a sample of 158 participants our design was adequately powered to detect a small/medium effect size (*w* = 0.28). Our testing took place in government-subsidized summer day camps, and we included all children within the predetermined age range of 4–10 years whose parents’ provided permission and who gave their assent. Parental consent was obtained prior to the session and child assent was obtained at the beginning of each session. This procedure was approved as minimal risk by the Research Ethics Board at (Blinded for review).

### Design

This study included two factors that varied between participants; Decision Time condition (Fast, Slow) and Inequity Type (Advantageous: 4 self/ 1 partner, Disadvantageous: 1 self/ 4 partner), and one within participant factor; Distribution (Equal, Unequal). Participants were randomly assigned to pairs (age- and sex-matched) and roles in the game (actor who made decisions, recipient who was passive). Participants were assigned to one of two Decision Time conditions. In the Fast condition they were told to make their decisions as quickly as possible, whereas in the Slow condition they were asked to wait and think about their decision until the experimenter prompted them to respond after a 10-s delay. Inequity type was varied in the Inequity Game, which consisted of 6 equal (1 each) and 6 unequal test trials (either Advantageous or Disadvantageous, depending on condition), with order of equal and unequal trials varying randomly across the 12 trials. Thus, there were four possible Decision Time X Inequity Type conditions: Fast/Advantageous, Slow/Advantageous, Fast/Disadvantageous, or Slow/Disadvantageous.

### Materials

**Figure 1:**
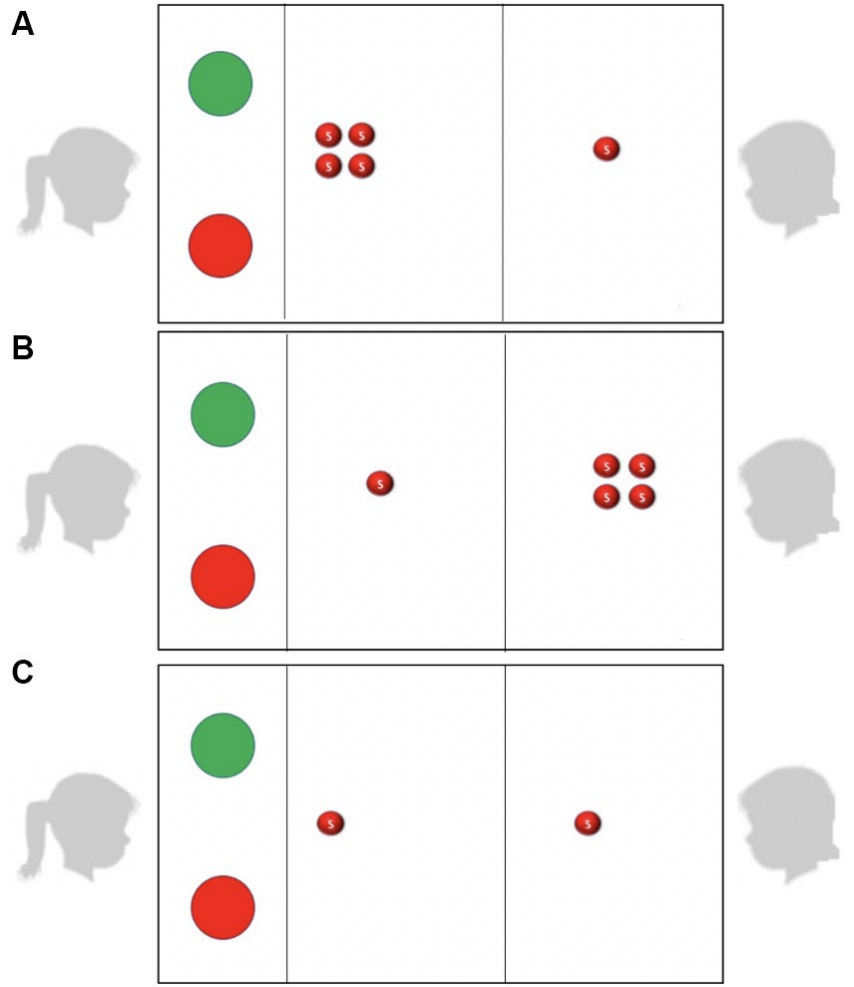
Examples of stimuli showing the allocation configuration for **(A)** advantageous, **(B)** disadvantageous and **(C)** equal trials. Actors sat facing the left side of the tablet, and recipients facing the right side. The buttons for the actor to accept or reject offers are in the left most panel of the figure, resources for the actors are in the middle panel and resources for the recipient are in the right most panel.

We employed a modified version of the child-friendly Inequity Game (developed by Blake and McAuliffe, 2011), that has been used to study inequity aversion across several studies (e.g., Blake and McAuliffe, 2011; Blake et al., 2015; Corbit et al., 2017, 2023b). The original Inequity Game is administered with a physical apparatus that allows the actor to accept or reject distributions of candies by pulling a red handle to reject and green handle to accept (but see Ahl et al., 2023 for validation of an online version). In the current study we were specifically concerned about controlling the decision times that children had to process and decide how to respond to a distribution. Thus, we presented children with distributions on an Apple iPad tablet where the allocations for actor and recipient were depicted with images of Skittles (see Figure 1). The actor and recipient were asked to sit across from each other, and the tablet was oriented so that the resources for the actor were on the side closest to them and the resources for the recipient were on the side close to them (see Figure 1). Both the actor and recipient could see the resources allocated to both sides while the decision slides were displayed. Once the allocation was presented the actor indicated their decision to accept or reject the offer by pushing a red button (reject) or a green button (accept) on the tablet. When an offer was accepted, the experimenter distributed candies to the bowls of actor and the recipient, matching the distribution represented on the tablet, counting out the number of candies each received and saying these were “candies they could take home” after the game. When an offer was rejected, the experimenter stated that neither child received any candies. At the beginning of the procedure participants were asked if they liked Skittles; one participant reported that they did not like skittles and their data was excluded from analysis.

### Procedure

#### Practice phase

After being paired with another child, participants were introduced to the modified Inequity Game. The actor was asked to sit on the side of the table where they could access the red and green buttons on the tablet that would be used to accept or reject offers in the game. The recipient was asked to sit across from the actor where they could both see the allocations on the table. Next, the experimenter demonstrated, in counterbalanced order across participants, what would happen when the actor pushed the red button to reject an offer and the green button to accept an offer. Participants were instructed to avoid talking to each other during the game and not to begin eating their candies until after the game was complete. After the experimenter had demonstrated the consequences of pushing each button the actor was given a comprehension check to verify that they understood the function of each button; all participants passed the comprehension check. Next, the actor received three practice trials to become familiar with making decisions in the game. The order of the practice trials was counterbalanced, and practice trials consisted of an equal distribution (1 each), an advantageous distribution (1 self / 0 recipient) and a disadvantageous distribution (0 self / 1 recipient). If the actor chose to either accept or reject all three practice trials, they received an extra equal trial and were asked to push the opposite button to ensure they understood that they could choose either button.

#### Experimental phase

In the experimental phase the initial prompt varied according to whether the participant was in a Fast or the Slow condition. In the Fast conditions participants were told to make their decision as quickly as possible. When the participant was ready the experimenter advanced the stimulus slide to the first allocation and the actor made their decision by pressing either the red or green button. Participants were reminded before each subsequent trial to make their decision as quickly as possible. In the Slow conditions participants were asked to wait and think about their answer and told that the experimenter would let them know when they could tell them their decision (after 10 s). After a 10-s delay the experimenter told the actor they could make their decision. Before each trial the experimenter reminded the actor to hold their response until the experimenter was ready. When the actor made their decision by pushing the red or green button, the PowerPoint presentation advanced to a blank slide. Following the actor’s decision on all test trials, the experimenter distributed the corresponding number of candies into the children’s bowls and stated the amount each participant would receive (e.g., “you get four and you get one” or “you get one and you get one”). After allocating the candies the experimenter gave the prompt that corresponded to the assigned Decision Time condition, then advanced the stimulus slide to the next distribution. This procedure was repeated for each of 12 trials.

### Coding and statistical analyses

The primary outcome variable was whether children accepted or rejected allocations across trials in the modified Inequity Game (accept coded as 0, reject coded as 1). Data and analysis code are available at: https://osf.io/7g6mp/?view_only=6cf39cb2da9d498bacb221352d7392b1. Children’s decisions were live coded and then later coded from video by an independent coder to ensure reliability. Disagreements between live coding and video coding were rare (Cohen’s *κ* = 0.98) and any disagreements identified during reliability coding were resolved by the corresponding author checking from video. We also coded from video the actors’ response time from the onset of the decision slide presentation until they pushed a button to accept or reject allocations. Because we manipulated decision time across the Fast and Slow conditions, reaction time was used as a manipulation check, rather than a predictor variable. We used R software (Version 3.5.1) and the R Studio application (Version 1.2.5019) to conduct all statistical analyses (R Core Team, 2019; RStudio Team, 2019). Decision data were analyzed using General Linear Mixed Models (GLMMs) with a binary response term (reject = 1, accept = 0). Mixed models were run using function ‘glmer’ from the R package ‘lme4’ (Bates et al., 2012). Participant identity (ID) was fit as a random effect to control for repeated measures.

Our primary hypotheses focused on the influence of Decision Time condition on children’s inequity aversion, thus we created a full model that included the interaction between Decision Time (Fast, Slow), Distribution (Equal, Unequal), Inequity Type (Advantageous, Disadvantageous) and Age (continuous between 4 and 10 years). Following previous research (e.g., Blake et al., 2015) we considered that children displayed an aversion to inequity when they rejected unequal distributions significantly more often than equal distributions. The statistical significance of the full model was determined by comparing its fit with that of the null model comprising the random effect term (ID) and control factors of gender and trial number (1–12), using a likelihood ratio test (LRT). The full model was a better fit to the data than the null model (LRT, χ^2^_3_ = 484.68, *p* < 0.001). Given that we made separate predictions for advantageous and disadvantageous inequity aversion, we also tested whether Inequity Type (Advantageous, Disadvantageous) differentially influenced children’s probability of rejecting allocations (Corbit et al., 2017). The full model including Inequity Type was a better fit to the data than the reduced model where this term was dropped (LRT, χ^2^_8_ = 21.75, *p* < 0.01), thus advantageous and disadvantageous inequity aversion types were analyzed separately in subsequent analyses. Once again, gender and trial number were included as control factors in both the advantageous and disadvantageous models. In subsequent models, *p*-values for individual predictors were calculated from likelihood ratio tests comparing the full model with their respective reduced models (R function ‘drop1’), non-significant interactions were dropped to reliably interpret the lower-level interactions and main effects. Figures were created in R using the package ‘ggplot2’ (Wickham, 2016).

## Results

### Advantageous inequity aversion

**Figure 2:**
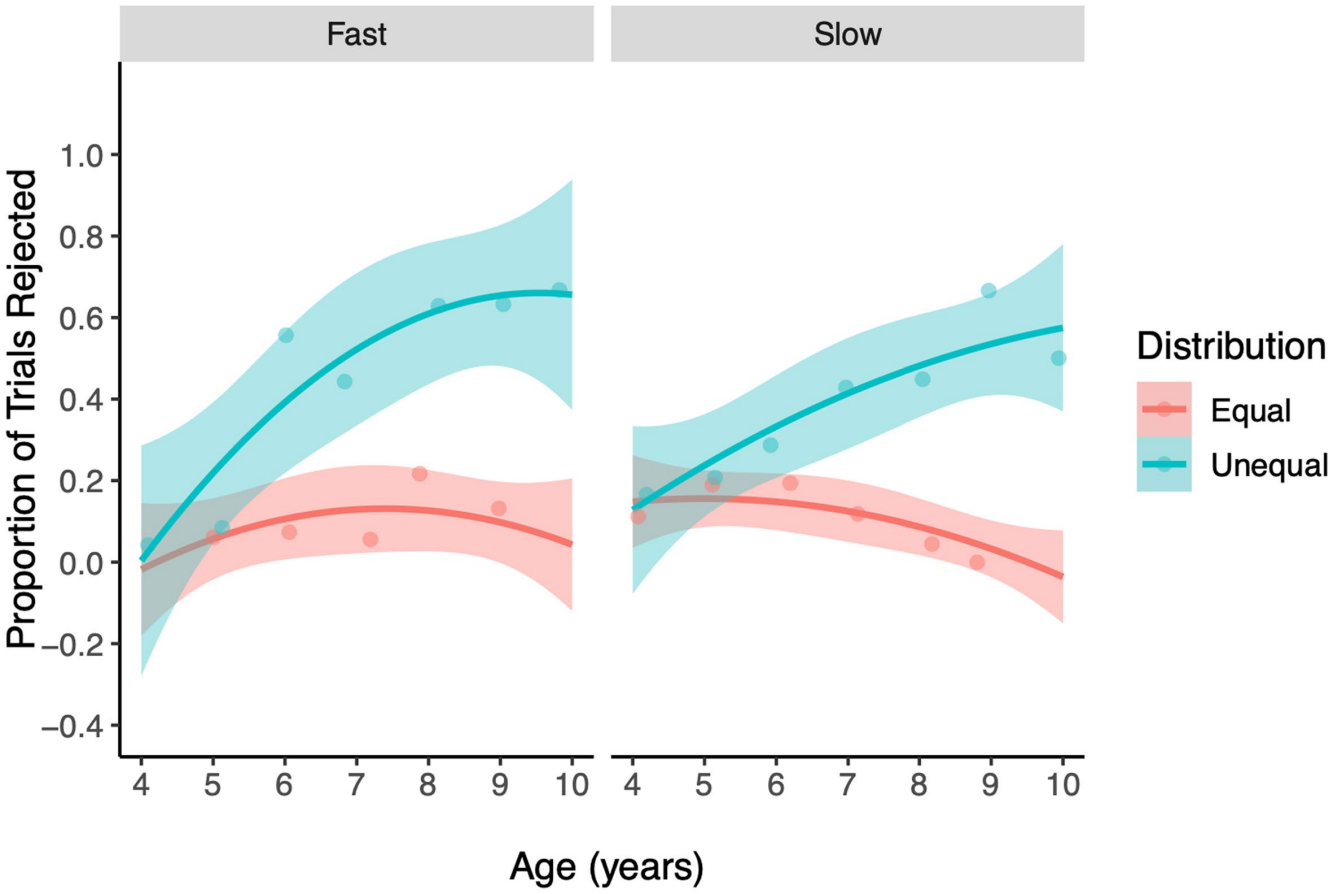
The regression lines represent the probability of rejecting offers in the Inequity Game for the Advantageous inequity type, plotted over Age (years), facetted by Decision Time (Fast, Slow). Ribbons show 95% confidence intervals. The jitter points (dots) represent the proportion of rejections at each level of Age, Distribution and Decision Time.

Our primary question was whether the development of advantageous inequity aversion, evidenced by a higher proportion of rejection on unequal compared to equal trials, was impacted by Decision Time condition and how this relation developed over age. The three-way interaction between Decision Time, Age, and Distribution was a significant predictor of children’s rejection of allocations (LRT, χ^2^_1_ = 4.48, *p* = 0.03, Figure 2, Supplementary Table S1). A significant three-way interaction suggests that the development of inequity aversion (age-related change in the relative rate of rejection of equal and unequal trials) differed across the Decision Time conditions. To explore how the development of children’s Advantageous inequity aversion differed across the Fast and Slow conditions we created a plot of the predicted values of this relation with a 95% confidence interval surrounding the regression lines. We consider the age at which children begin to display inequity aversion to be the point at which the confidence intervals around the regression lines predicting rejection rate are higher for unequal than equal trials and no longer overlap. Figure 2 reveals that children show a similar age of emergence, around 6 years of age, in both the Fast/Advantageous condition and the Slow/Advantageous condition. Furthermore, Figure 2 reveals that the significant three-way interaction is likely driven by a greater age-related increase in children’s rejection of unequal trials compared to equal ones in the Fast condition versus the Slow condition, though in both conditions children become increasingly more likely to reject unequal offers than equal offers with age. This interpretation is supported through a supplementary analysis treating age as a categorical variable (Age Group: 4–5, 6–7, and 8–10), which once again revealed a significant 3-way interaction (LRT, χ^2^_1_ = 4.48, *p* = 0.03, Supplementary Figure S1, Supplementary Table S2), with *post hoc* analyses revealing a significant Condition by Distribution interaction at the Age Group level of 6–7 years (LRT, χ^2^_1_ = 7.32, *p* < 0.01), a marginal effect at 8–10 years (LRT, χ^2^_1_ = 3.71, *p* = 0.054) and a non-significant effect at the level of 4–5 years (*p* = 0.70). The main effect of Trial number was not a significant predictor of rejections (LRT, χ^2^_11_ = 16.97, *p* = 0.11), nor was the main effect of Gender (*p* > 0.90). Overall, the developmental increase in advantageous inequity aversion was greater when children’s decisions were made under time pressure compared to when they were made after a delay.

### Disadvantageous inequity aversion

**Figure 3:**
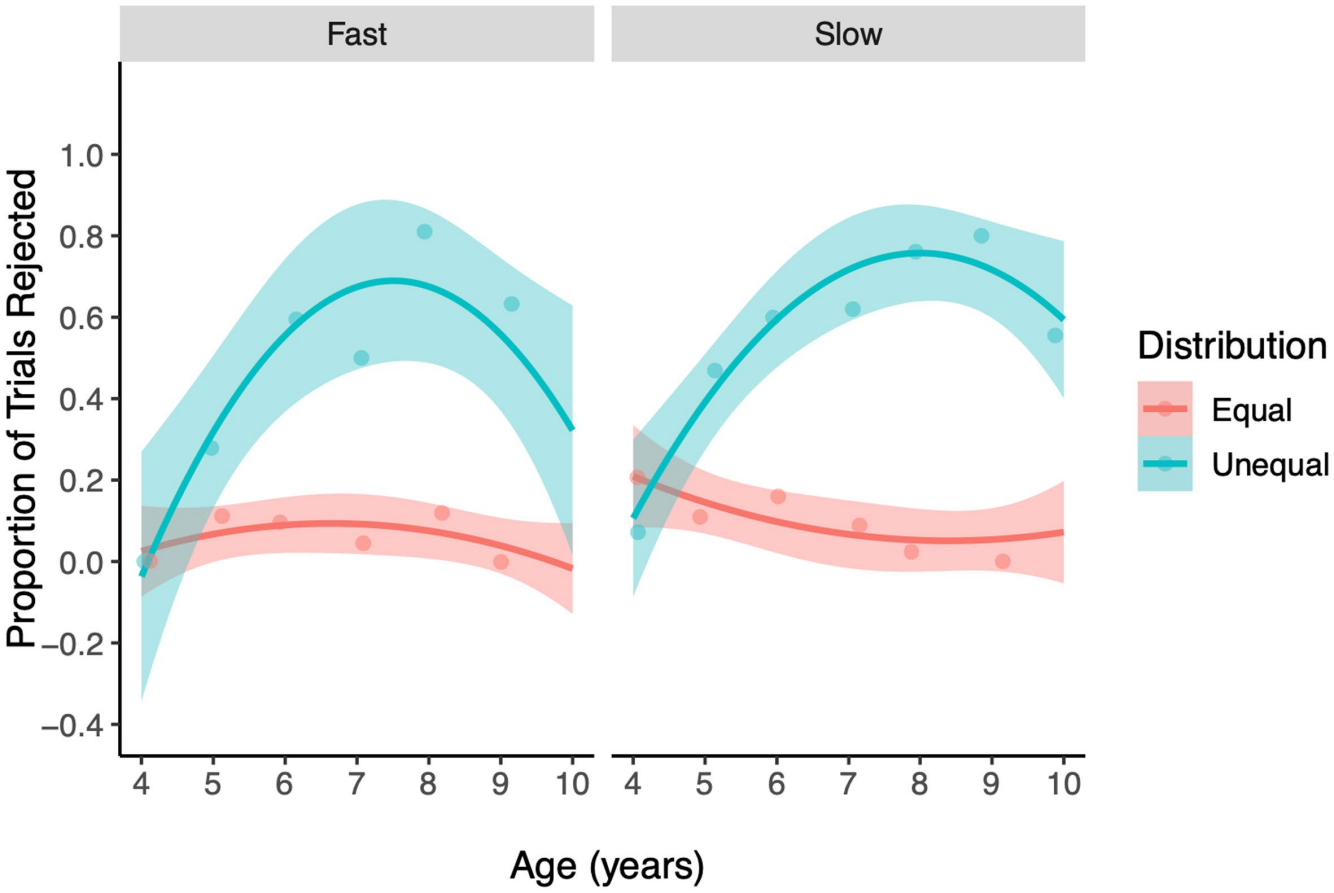
The regression lines represent the probability of rejecting offers in the Inequity Game for the Disadvantageous inequity type, plotted over Age (years), facetted by Decision Time (Fast, Slow). Ribbons show 95% confidence intervals. The jitter points (dots) represent the proportion of rejections at each level of Age, Distribution and Decision Time.

**Figure 4:**
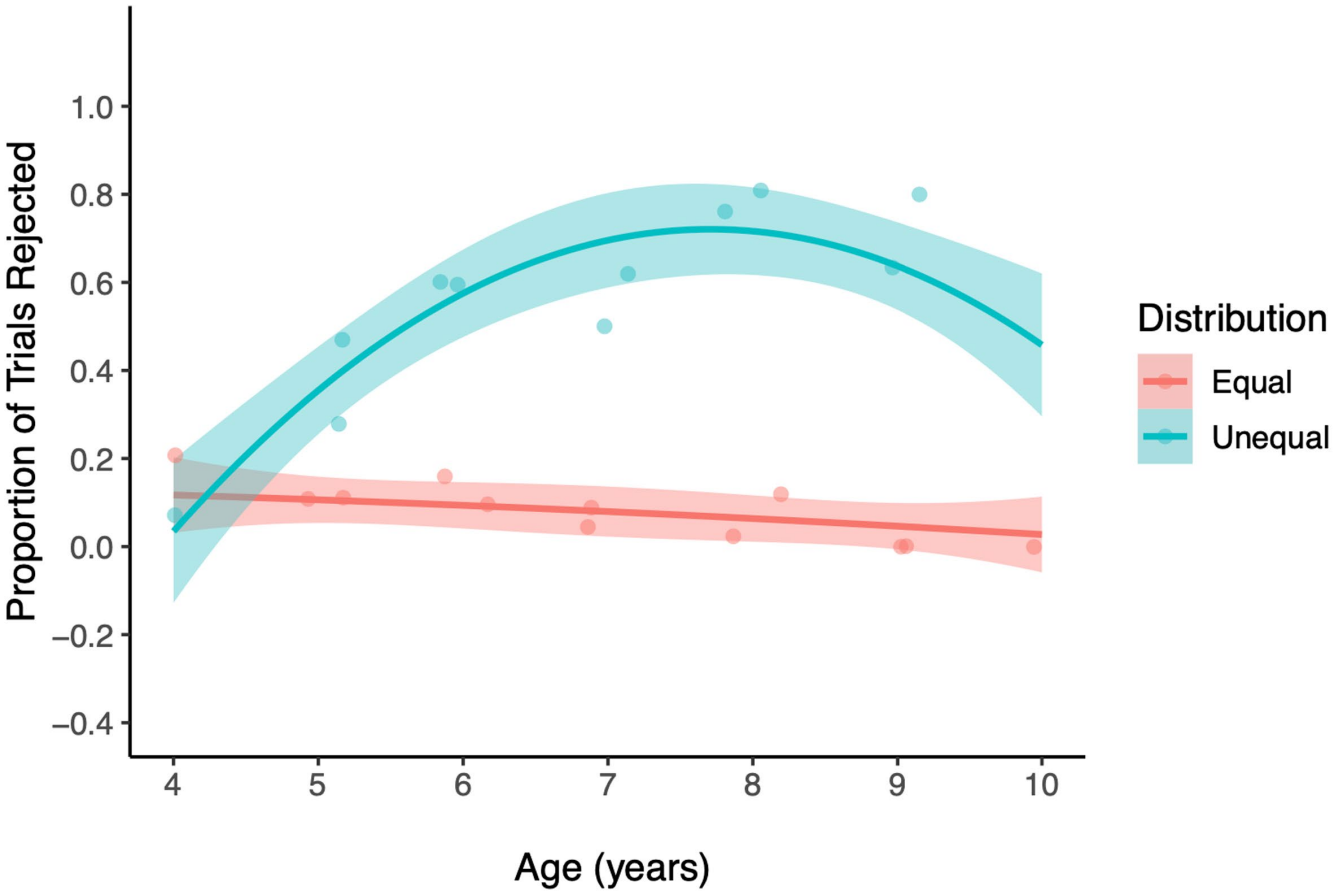
The regression lines represent the probability of rejecting offers in the Inequity Game for the Disadvantageous inequity type, plotted over Age (years). Ribbons show 95% confidence intervals. The jitter points (dots) represent the proportion of rejections at each level of Age and Distribution.

Once again, we were primarily interested in whether the development of disadvantageous inequity aversion was impacted by decision time and the development of this relation. The three-way interaction between Decision Time, Age, and Distribution was not a significant predictor of children’s rejection of allocations (LRT, χ^2^_2_ = 0.26, *p* = 0.61, Figure 3, Supplementary Table S1), and was dropped from the model. The reduced model was comprised of a significant 2-way interaction between Age and Distribution (LRT, χ^2^_1_ = 37.59, *p* < 0.001, Figure 4) and two non-significant interactions between Decision Time and Distribution (LRT, χ^2^_2_ = 0.001, *p* = 0.97), and Age and Decision Time (LRT, χ^2^_1_ = 0.016, = 0.90). The main effects of Gender and Trial number were not statistically significant (*p* > 0.2). Figure 3 reveals that disadvantageous inequity aversion was observed around 5 years of age in both conditions and tended to increase with age until middle childhood (around 8 years of age), when it began to show a small decline, a trend that was similar across the two Decision Time conditions (see Supplementary Figure S2, Supplementary Table S2 for equivalent findings with Age Group analysis). The overall development of children’s disadvantageous inequity aversion captured by the significant Age X Distribution interaction is depicted in Figure 4.

## Discussion

The primary goal of our study was to assess the relative influence of intuitive and reflective decision processes on the development of children’s fairness behavior. We were specifically interested in the impact of these decision processes on two forms of fairness, advantageous and disadvantageous inequity aversion. In both the Fast and Slow conditions children rejected unequal advantageous offers more often than equal offers beginning between 6 and 7 years of age, and this tendency increased with age. Importantly, we found that the age-related increase in advantageous inequity aversion was greater in the Fast condition relative to the Slow condition. This pattern provided partial support for H1; although the age of emergence was similar across Fast and Slow conditions, the age-related increase in advantageous inequity aversion was greater in the Fast condition compared to the Slow condition. We failed to find evidence for H2; that deliberate decisions would allow children to inhibit self-maximizing tendencies and increase costly fairness. On disadvantageous trials we did not find that decision time impacted children’s disadvantageous inequity aversion. In both the Fast and Slow conditions, children rejected unequal offers more often than equal ones from 5 years of age, a tendency that increased with age until around 8 years, at which point rejection of unequal offers began to decline. This pattern did not provide evidence in support of either an increase in generosity (H3) or reactive aggression (H4) stemming from intuitive decision processes. Thus, taken together our findings reveal that recruiting intuitive decision processes in fairness decisions may lead to a more robust expression of children’s tendency to reject resource distributions that give them a relative advantage over a peer.

To our knowledge this was the first study that directly tested the impact of intuitive and deliberate decision processes on the development of inequity aversion. There are several related studies that may appear to provide contradictory evidence, but which have important methodological differences. In one study, Chajes et al. (2022) presented children (4–9 years of age) with a forced choice sharing decision between an equal (2 resources each) or selfish (3 for self, 1 for recipient) distribution. Younger children (4–6 years of age) rarely chose the equal option (~20% of the time), but when they did, equal decisions tended to be slower compared to selfish decisions. However, decision time did not predict decisions for older children (7–9 years of age), who were more likely to choose the equal option (>60% of the time). Two methodological differences could account for the divergent pattern of results between the current study and Chajes et al. (2022). First, as noted in the introduction, fair decisions in the Chajes et al. (2022) paradigm were also generous because they increased the number of resources given to the recipient (2 as opposed to 1), whereas fair decisions in the current study were non-generous because rejecting unequal offers reduced the payoff for both the actor and recipient. Meta-analytic evidence from adults suggests that while altruistic giving was not positively impacted by intuitive relative to deliberative decision processes, the tendency to reject unfair offers in the ultimatum game was (Fromell et al., 2020; Capraro, 2024), which could provide some insight into the differences observed in the current study compared to those of Chajes et al. (2022). A second methodological difference relates to decision time. Decision time varied naturally in the Chajes et al. (2022) study, whereas decision time was experimentally manipulated to be under time pressure (Fast conditions) or after a 10-s delay (Slow conditions) in the current study. It is possible that experimentally manipulating decision time in the current procedure may have increased the impact of decision time on fairness preferences compared to procedures where decision time varies naturally across cooperative decisions. Indeed, it has been proposed that correlational designs, where response time varies naturally, correspond to decision conflict rather than intuitive or deliberate decision processes (Evans and Rand, 2019). It is also worth noting that previous studies that have revealed evidence for intuitive cooperation in children’s resource allocation decisions manipulated decision time (Plötner et al., 2021; Corbit et al., 2023a), whereas those that did not find evidence for an intuitive effect allowed decision times to vary naturally (Blake and McAuliffe, 2011; Chajes et al., 2022). The role of intuitive decisions in the development of cooperative behaviors is a relatively new area of empirical inquiry and so more evidence is needed before firm conclusions can be made, especially studies that attempt to replicate the effects and meta-analyses when sufficient data is available (see Rand, 2016; Bouwmeester et al., 2017; Fromell et al., 2020; Capraro, 2024).

An additional methodological feature of the current study that might have impacted our results is that children’s decisions were made on a tablet where the distribution of candies were represented symbolically as cartoon skittles. Although real Skittles were distributed on every trial that participants accepted an offer, it is possible that symbolic distancing affected decisions on the tablet compared to the apparatus employed in previous studies (e.g., Blake et al., 2015). In a study using the classic ‘less is more’ task, which requires pointing to a smaller reward in order to receive a larger reward, 3-year-old children’s performance improved significantly when the rewards were symbolic representations of candies compared to real candies (Carlson et al., 2005). These findings were interpreted to suggest that inhibiting the desire to point to a larger, more enticing, reward was easier when the rewards were represented symbolically. However, studies that have examined the impact of symbolic distancing on fairness decisions have produced mixed results. Ebersbach et al. (2022) found that children (4–6 years old) were more likely to reject unequal offers when they were represented symbolically (tokens vs. stickers). In contrast, Ahl et al. (2023) found that children were less likely to reject advantageous offers in a token-based online compared to an in-person real candy version of the inequity game. If symbolic distancing reduced the inhibitory demands of rejecting unequal distributions in the inequity game in the current study, it would be more likely that reduced inhibitory demands would wash out any benefit from employing reflective decision processes, rather than enhance the benefit of intuitive decisions. Thus, it is unlikely that symbolic distancing can account for increased fairness decisions under intuitive conditions in the current study. Nevertheless, additional research is needed to tease apart the potential role of symbolic distancing on the relative impact of intuitive and reflective decision processes on costly cooperative behavior.

### Limitations

An important limitation of the current study is that we only investigated the impact of intuitive decisions of children’s fairness preferences amongst children living in a single Canadian community. Past research has revealed considerable diversity in the development of fairness across diverse cultural contexts, particularly in the development of advantageous inequity aversion (Blake et al., 2015; Paulus, 2015; Kajanus et al., 2019; Li et al., 2022). While we are not aware of any studies that have investigated intuitive fairness across cultures, one study examined the impact of decision time on cooperative decisions amongst adults in a public goods game in the USA and India (Nishi et al., 2017). In this study, adults in the USA were more likely to cooperate when decisions were made quickly, whereas adults in India made cooperative decisions more slowly than selfish ones. This pattern suggests that cultural differences may exist in the impact of intuitive and reflective decisions on cooperative behaviors and thus, future research should examine the development of intuitive fairness across diverse societies, particularly where different fairness norms exist.

Another important area for future research will be to extend investigations of intuitive fairness to older age ranges. There is evidence to suggest that a developmental shift occurs during middle childhood when children’s aversion to disadvantageous inequity begins to decrease as children become more likely to allow their peers to gain a relative advantage. We observed the emergence of this trend in older participants in the current study (see Figure 4, 8–10 years of age) but did not observe a significant difference in children’s age-related change in inequity aversion between the Fast and Slow conditions. It is possible that with an extended age range we might have observed a difference across Fast and Slow decision contexts in the age of emergence of the trend toward diminishing disadvantageous inequity aversion.

## Conclusion

This is the first study to examine the impact of intuitive and reflective decision processes on children’s aversion to advantageous and disadvantageous inequity. We found that fast, intuitive decisions led to a greater age-related increase of advantageous inequity aversion compared to decisions made after a time delay. In contrast, we did not find evidence that disadvantageous inequity aversion was impacted by decision time. These findings suggest that recruiting intuitive decision processes might strengthen the emergence of advantageous inequity aversion during middle childhood.

## Data availability statement

The datasets presented in this study can be found in online repositories. The names of the repository/repositories and accession number(s) can be found at: https://osf.io/7g6mp/?view_only=6cf39cb2da9d498bacb221352d7392b1.

## Ethics statement

The studies involving humans were approved by the Research Ethics Board at St Francis Xavier University. The studies were conducted in accordance with the local legislation and institutional requirements. Written informed consent for participation in this study was provided by the participants’ legal guardians/next of kin.

## Author contributions

JC: Writing – review & editing, Writing – original draft, Visualization, Validation, Supervision, Software, Resources, Project administration, Methodology, Investigation, Funding acquisition, Formal analysis, Data curation, Conceptualization.
