# Supplementary material for: Time pressure increases children’s aversion to advantageous inequity
Source: Front Psychol. 2024 Jun 5;15:1390741. doi: 10.3389/fpsyg.2024.1390741 (PMC11186461; doi:10.3389/fpsyg.2024.1390741)
Supplement: Supplementary file 1 [file Table_1.DOCX]

**Supplementary Online Material: Time pressure increases children’s aversion to advantageous inequity**

**Table 1.** Model outputs and age as a continuous variable.

|  | **AI Model Full** | **AI Model Reduced** | **DI Model Full** | **DI Model Reduced** |
| --- | --- | --- | --- | --- |
| (Intercept) | -5.42^***^ | -5.11^***^ | -1.62 | -0.56 |
|  | (1.51) | (1.44) | (1.57) | (1.18) |
| Age | 0.32 | 0.34 | -0.31 | -0.38^*^ |
|  | (0.20) | (0.19) | (0.23) | (0.18) |
| Gender (Male) | -0.01 |  | -0.03 |  |
|  | (0.32) |  | (0.30) |  |
| Trial (2) | 0.58 |  | 0.76 |  |
|  | (0.46) |  | (0.43) |  |
| Trial (3) | 1.38^**^ |  | 0.92 |  |
|  | (0.48) |  | (0.47) |  |
| Trial (4) | 0.49 |  | 0.92^*^ |  |
|  | (0.48) |  | (0.44) |  |
| Trial (5) | 0.55 |  | 0.90^*^ |  |
|  | (0.48) |  | (0.45) |  |
| Trial (6) | -0.44 |  | 0.38 |  |
|  | (0.56) |  | (0.47) |  |
| Trial (7) | 0.48 |  | 0.47 |  |
|  | (0.48) |  | (0.45) |  |
| Trial (8) | 0.04 |  | 0.25 |  |
|  | (0.47) |  | (0.43) |  |
| Trial (9) | 0.17 |  | 0.61 |  |
|  | (0.50) |  | (0.45) |  |
| Trial (10) | 0.45 |  | 1.12^*^ |  |
|  | (0.48) |  | (0.45) |  |
| Trial (11) | 0.40 |  | 0.53 |  |
|  | (0.48) |  | (0.45) |  |
| Trial (12) | 0.08 |  | 0.23 |  |
|  | (0.50) |  | (0.45) |  |
| Distribution (Unequal) | -0.21 | 0.04 | -2.13 | -2.49^**^ |
|  | (1.45) | (1.43) | (1.53) | (0.95) |
| Condition (Slow) | 5.89^**^ | 5.85^**^ | 1.28 | 0.42 |
|  | (1.98) | (1.95) | (1.92) | (1.24) |
| Dist (Uneq): Cond (Slow) | -4.47^*^ | -4.53^*^ | -0.94 | -0.05 |
|  | (2.00) | (1.97) | (1.91) | (0.41) |
| Age: Cond (Slow) | -0.85^**^ | -0.84^**^ | -0.14 | 0.01 |
|  | (0.29) | (0.29) | (0.29) | (0.18) |
| Age: Dist (Uneq) | 0.39 | 0.34 | 0.77^**^ | 0.82^***^ |
|  | (0.20) | (0.20) | (0.23) | (0.14) |
| Age:Dist (Uneq):Cond (Slow) | 0.60^*^ | 0.61^*^ | 0.15 |  |
|  | (0.29) | (0.29) | (0.29) |  |
| AIC | 787.04 | 780.02 | 936.23 | 923.83 |
| BIC | 887.27 | 822.98 | 1039.63 | 963.22 |
| Log Likelihood | -372.52 | -381.01 | -447.12 | -453.92 |
| Num. obs. | 874 | 874 | 1016 | 1016 |
| Num. groups: ID | 73 | 73 | 85 | 85 |
| Var: ID (Intercept) | 1.06 | 1.03 | 1.09 | 1.04 |
| ^***^p < 0.001; ^**^p < 0.01; ^*^p < 0.05 | | | | |

**Table 2.** Model outputs and age as a categorical variable.

| Statistical models | | | |
| --- | --- | --- | --- |
|  | **AI Model Full** | **DI Model Full** | **DI Model Reduced** |
| (Intercept) | -3.53^***^ | -2.72^***^ | -2.54^***^ |
|  | (0.68) | (0.55) | (0.38) |
| Age Group (6_7) | 0.54 | -0.28 | -0.22 |
|  | (0.87) | (0.74) | (0.46) |
| Age Group (8_10) | 1.57^*^ | -0.37 | -1.04 |
|  | (0.80) | (0.77) | (0.53) |
| Distribution (Unequal) | 0.56 | 1.20^*^ | 1.17^***^ |
|  | (0.77) | (0.55) | (0.34) |
| Condition (Slow) | 1.65 | 0.71 | 0.41 |
|  | (0.84) | (0.70) | (0.27) |
| Distribution (Unequal):Condition (Slow) | -0.36 | -0.05 |  |
|  | (0.93) | (0.70) |  |
| Age Group (6_7): Condition (Slow) | -0.60 | 0.08 |  |
|  | (1.08) | (0.95) |  |
| Age Group (8_10): Condition (Slow) | -3.75^**^ | -1.32 |  |
|  | (1.24) | (1.09) |  |
| Age Group (6_7): Distribution (Unequal) | 2.47^**^ | 2.01^**^ | 1.74^***^ |
|  | (0.94) | (0.75) | (0.46) |
| Age Group (8_10): Distribution (Unequal) | 2.03^*^ | 2.68^***^ | 3.19^***^ |
|  | (0.88) | (0.78) | (0.55) |
| Age Group (6_7): Distribution (Uneq): Condition (Slow) | -1.42 | -0.46 |  |
|  | (1.15) | (0.95) |  |
| Age Group (8_10): Distribution (Uneq): Condition (Slow) | 1.92 | 1.04 |  |
|  | (1.30) | (1.11) |  |
| AIC | 773.89 | 924.91 | 917.39 |
| BIC | 835.94 | 988.92 | 956.78 |
| Log Likelihood | -373.94 | -449.46 | -450.69 |
| Num. obs. | 874 | 1016 | 1016 |
| Num. groups: ID | 73 | 85 | 85 |
| Var: ID (Intercept) | 0.85 | 0.86 | 0.84 |
| ^***^p < 0.001; ^**^p < 0.01; ^*^p < 0.05 | | | |

**Figure S1.** The proportion of rejections in the Inequity Game for the Advantageous inequity type separated by Distribution type (Equal, Unequal), plotted over Age Groups (4_5, 6_7, and 8_10 years), facetted by Decision Time (Fast, Slow). Error bars show 95% confidence intervals.

**Figure S2.** The proportion of rejections in the Inequity Game for the Disadvantageous inequity type separated by Distribution type (Equal, Unequal), plotted over Age Groups (4_5, 6_7, and 8_10 years), facetted by Decision Time (Fast, Slow). Error bars show 95% confidence intervals.
